# Fiberoptic bronchoscopic treatment of blood aspiration and use of sugammadex in a patient with epistaxis: A case report

**DOI:** 10.1097/MD.0000000000010428

**Published:** 2018-04-13

**Authors:** Taeha Ryu, Dong Hyuck Kim, Sung Hye Byun

**Affiliations:** Department of Anesthesiology and Pain Medicine, School of Medicine, Catholic University of Daegu, Daegu, Republic of Korea.

**Keywords:** blood aspiration, epistaxis, fiberoptic bronchoscopy, sugammadex

## Abstract

**Rationale:**

In patients with oropharyngeal and nasopharyngeal bleeding, blood aspiration can make airway management difficult and lead to severe pulmonary complications.

**Patient concerns:**

A 44-year-old male patient with recurrent epistaxis underwent surgery for hemostasis. The patient aspirated blood through the endotracheal tube when he hiccupped during the surgery.

**Diagnosis:**

The patient was diagnosed with blood aspiration after intraoperative fiberoptic bronchoscopy revealed a blood clot and viscous mucus in the airways, but no sign of active bleeding.

**Interventions:**

Tracheobronchial suctioning and irrigation with normal saline was performed through the bronchoscope to remove the aspirated blood clot. Prior to emergence from anesthesia, sugammadex was administered to induce complete neuromuscular recovery and enable the patient to cough up any blood remaining in the airways.

**Outcomes:**

The patient was successfully extubated and fully recovered with no complications.

**Lessons:**

Blood aspiration due to oropharyngeal or nasopharyngeal bleeding can be diagnosed and treated by tracheobronchial suctioning via fiberoptic bronchoscopy. In addition, sugammadex can enable patients to recover spontaneous breathing, facilitate extubation, and enable patients to cough up any blood remaining in the airways.

## 1 Introduction

Perioperative airway management can be challenging in patients with oropharyngeal and nasopharyngeal bleeding. Under general anesthesia, loss of consciousness and muscle relaxation result in suppression of airway reflexes, increasing the risk of blood aspiration. The risk of pulmonary complications due to blood aspiration is reportedly lower than in cases in which acidic gastric contents are aspirated.^[1]^ However, animal experiments have shown that blood aspiration can cause a significant intrapulmonary shunt and impair host defense mechanisms, resulting in increased susceptibility to pulmonary bacterial infection.^[2]^ Moreover, when a large volume of blood is aspirated, blood clots can cause airway obstruction, atelectasis, hypoxemia, and even death.^[3–5]^ Here, we report the case of a patient who underwent surgery under general anesthesia to control bleeding due to recurrent epistaxis, during which the patient aspirated blood intraoperatively. We promptly performed tracheobronchial suctioning using a fiberoptic bronchoscope (FOB) and successfully removed the aspirated blood clot. We also administered sugammadex to induce complete reversal of the neuromuscular blockade (NMB), which enabled the patient to be successfully extubated and cough up any blood remaining in the airways. The use of sugammadex may have prevented the development of more severe pulmonary complications, enabling a full recovery.

## 2 Case report

A 44-year-old male patient (170 cm, 68 kg) who had previously undergone surgical excision of a nasal polyp 4 years prior to admission, presented at our hospital's emergency room (ER) complaining of recurrent epistaxis. Three weeks prior, following severe coughing, the patient experienced bleeding from the nose and mouth at a volume sufficient to fill a paper cup (approximately 150 mL). However, when the patient visited a local clinic, the bleeding had already stopped and he was discharged without any specific treatment. One week prior to admission, the patient presented to the emergency room (ER) when the bleeding recurred; however, endoscopy revealed no active bleeding in the nasal cavity, and the site of the previous surgery was clear. Esophagogastroduodenoscopy and colonoscopy were performed to rule out gastrointestinal bleeding, which revealed no source of bleeding. The patient's hemoglobin (Hb) level was 8.6 g/dL at that time, which recovered to 11.9 g/dL after transfusion of 2 units of packed red blood cells (PRBC). The patient was discharged home without any further bleeding.

**Figure 1:**
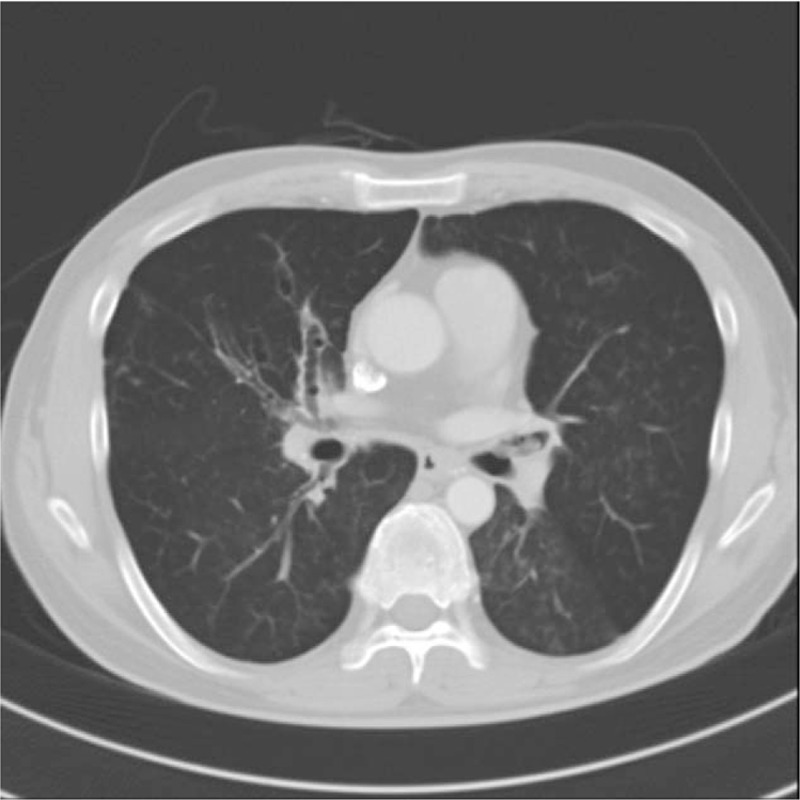
Chest computed tomography performed after the patient coughed up blood showed a suspected blood clot in the left upper lobe bronchus, secretions in both lower bronchi and the left upper lobe bronchus, as well as multiple centrilobular nodules and patchy consolidation in both lungs.

The patient returned to our ER following another episode of epistaxis, again with a blood loss of about 150 mL. Angiography of both external carotid arteries was performed, which revealed no obvious vascular malformation or arterial bleeding. Nevertheless, the patient continued to experience sporadic epistaxis, and so emergency surgery was planned for hemostasis. Once the patient arrived to the operating theater, standard monitoring devices were applied, including electrocardiography, pulse oximetry, and a noninvasive blood pressure cuff. Total intravenous anesthesia was induced and maintained using propofol and remifentanil, and intravenous rocuronium 50 mg was administered for muscle relaxation. The patient was intubated with a 7.5-mm endotracheal tube (ETT) with a tapered-shaped cuff (TaperGuard, Mallinckrodt, Covidien, Athlone, Ireland). At the time of intubation, the oral cavity was clear with no blood and the laryngeal view was Cormack–Lehane grade I. After anesthesia induction and intubation, the surgeon endoscopically examined the nasal cavity, but no active bleeding was detected, and only traces of bleeding remained, such as blood clots. The nasal cavity was packed with gauze. Capnography showed an obstructive pattern throughout the surgery. Fifty minutes after the initial administration of rocuronium, the patient hiccupped, and bright red blood regurgitated back through the ETT. An additional dose of rocuronium 50 mg was administered immediately, and a small volume of blood was suctioned out of the endotracheal tube. At that time, rales and wheezing were heard on chest auscultation, which had not been heard preoperatively. The patient had a history of bronchiectasis diagnosis 3 years earlier; therefore, a FOB was inserted through the ETT after surgery to check for endobronchial bleeding. No active bleeding was observed, but blood clots and viscous mucus were found in both main bronchi, especially in the dependent portions; suction tubing was connected to the FOB and normal saline irrigation was performed to remove the blood clots and mucus. The likelihood of endobronchial bleeding seemed low, and we suspected pulmonary aspiration of nasal bleeding and oral secretions. Therefore, prior to emergence, we used a suction catheter to remove blood that had pooled in the oral cavity stopped administration of intravenous anesthetic. We then intravenously administered sugammadex 280 mg to induce complete reversal of the NMB, which was about 40 minutes after the last dose of rocuronium. Due to a mechanical defect in our train-of-four (TOF) monitor, we were unable to determine the exact degree of NMB. Although the degree of NMB was not expected to be deep, we wanted to maximize reversal for decreasing the incidence of residual paralysis and postoperative pulmonary complication,^[6]^ resulted from incomplete recovery of muscle relaxation. Acetylcholinesterase inhibitors, the most commonly used agents for NMB reversal, are known to be effective only after spontaneous recovery has begun, namely when the fourth twitch of TOF stimulation is detectable.^[6,7]^ Therefore, rather than choosing an acetylcholinesterase inhibitor, we chose 4 mg/kg of sugammadex, which is the recommended dosage for reversal of deep NMB, defined as no TOF response and a post-tetanic count of 1 to 2 twitches. After spontaneous respiration was restored, the patient began coughing and was extubated. As the patient began coughing, about 75 mL of blood, thought to be blood remaining in the bronchi and nasopharyngeal cavity, was regurgitated into the oral cavity. The patient was placed in the Trendelenburg position to facilitate drainage. The patient's peripheral oxygen saturation (SpO_2_) temporarily decreased to just over 90% while he was coughing, but his SpO_2_ returned to and remained at 98% with the administration of 5 L of oxygen by facemask. Because the volume of regurgitated blood gradually decreased and his coughing subsided, we elected not to reintubate the patient. Due to the risk of recurrent bleeding and asphyxia, we admitted the patient to the intensive care unit (ICU) for further observation. A postoperative complete blood count showed a Hb level of 9.1 g/dL, and the patient was transfused 2 units of PRBC. About 6 hours postoperatively, the patient suddenly began coughing and approximately 100 mL of blood was regurgitated into the oral cavity. At the time of coughing, the patient's SpO_2_ dropped to 90% and he complained of dyspnea. After a 10-minute coughing episode, oxygen 2 L/min was administered by nasal cannula to maintain an SpO_2_ at 95% to 98%, and the patient's dyspnea was alleviated. Chest computed tomography performed immediately after the coughing and bleeding stopped showed a suspected blood clot in the left upper lobe bronchus, secretions in both lower bronchi and the left upper lobe bronchus, as well as centrilobular nodules and patchy consolidation in both lungs (Fig. 1). The patient was encouraged to cough in order to remove any remaining blood clots and mucus from his airways. To minimize the chance of further bleeding, the patient was started on intravenous tranexamic acid 500 mg 3 times a day and vitamin K 10 mg once a day. A repeat Hb level was found to be 9.9 g/dL, which recovered to 11.3 g/dL after transfusion of 2 units of PRBC. Sporadically, dilute epistaxis was observed and old blood clots were expelled during coughing, but the volume of blood loss was very low and the patient's vital signs remained stable. The patient was discharged from the ICU on postoperative day 3. Subsequent endoscopy showed signs of blood trickling towards the posterior part of the right turbinates, suggesting posterior nasal bleeding. The patient was ultimately discharged home and has experienced no further bleeding or complications. Ethical approval was not required from the institutional review board as the clinical data were deidentified; however, the patient in this case provided written informed consent for the publication of this report.

## 3 Discussion

Perioperative pulmonary aspiration is a severe complication in anesthesiology. According to a recent analysis of the American Society of Anesthesiologists closed claims’ database,^[8]^ pulmonary aspiration is the third most common respiratory event related to anesthesia, following difficult intubation and inadequate ventilation. According to recently published data from the United Kingdom,^[5]^ pulmonary aspiration is the most common cause of airway related mortality during anesthesia. Pulmonary aspiration can present with a diverse spectrum of symptoms, from mild bronchial irritation to acute respiratory distress syndrome.^[9]^ Even apart from gastric contents, aspiration of various sizes of particulate matter can cause airway obstruction, atelectasis, hypoxemia, and pulmonary edema. Although the likelihood of pulmonary complications is known to be relatively low with blood aspiration,^[1]^ aspiration of a large volume of blood can cause airway obstruction, atelectasis,^[3]^ hypoxemia,^[4]^ and even death.^[5]^ One animal experiment using rats reported that intrapulmonary bacterial inactivation was significantly impaired 24 hours after intratracheal injection of blood.^[2]^ The presence of blood can provide a bacterial growth-promoting medium, and hemoglobin crystals and other blood components in the lungs inhibit bacterial phagocytosis. Therefore, blood aspiration can significantly increase a patient's susceptibility to bacterial infection.^[2]^ When blood is aspirated into the trachea, it is important to promptly remove the blood even if the patient shows no symptoms of hypoxemia.

Blood aspiration is known to be most closely associated with intraoral and otorhinolaryngologic surgery.^[10]^ Care is especially required when these patients undergo general anesthesia, due to loss of consciousness and protective airway reflexes. Furthermore, oropharyngeal fluid can be aspirated even after tracheal intubation. The ETT that we are currently using has a high-volume, low-pressure cuff; although the low-pressure cuff can reduce the risk of ischemic injury to the tracheal wall, longitudinal folds can form in the cuff when it is fully inflated, allowing fluids to leak around the ETT.^[11]^ Recently, tapered-shaped cuff designs have been increasingly used to minimize the formation of fluid channels and provide more secure sealing of the airway. Several studies have reported that tapered-shaped cuff designs more effectively reduce fluid leakage into the airway than conventional cylindrical cuff designs.^[12,13]^ Nevertheless, in vitro studies have found that tapered-shape cuffs cannot completely prevent fluid leakage around the cuff unless a positive end-expiratory pressure (PEEP) of at least 15 cm H_2_O is used.^[14,15]^ At our hospital, we do not routinely use PEEP, and in the present case, we cannot exclude the possibility that a large volume of nasal bleeding was aspirated intraoperatively. Therefore, surgeons and anesthesiologists should monitor for blood aspiration throughout the perioperative period, and thorough intratracheal and oropharyngeal suctioning should be applied.

When pulmonary aspiration is suspected or confirmed, the patient should immediately be placed in a head-down posture and the oral cavity and pharynx should be suctioned. If necessary, intubation should be performed followed by frequent endobronchial suctioning and carefully controlled ventilation to prevent aspiration into the airways. In patients who have already been intubated, as in the present case, use of an FOB can also be considered. In one report of blood aspiration caused by nasal bleeding during nasotracheal intubation, an FOB was used to directly verify aspirated blood in the lobar bronchi^[16]^; in a similar case, suction was applied to the FOB to effectively remove the aspirated blood.^[3]^ Suctioning through an FOB can remove aspirated materials in the distal bronchi that cannot be removed by blind airway suctioning. Another advantage of using an FOB is that it is possible to verify whether there is active endotracheal or endobronchial bleeding. In the present case, we used the FOB to confirm that there was no active endobronchial bleeding. Therefore, we suspected aspiration of blood from nasopharyngeal bleeding. If we had not used the FOB, the blood that the patient regurgitated during emergence could have been mistaken for hemoptysis, which would have prevented extubation and led to potentially unnecessary treatment. Therefore, in cases of endotracheal blood aspiration, the use of an FOB can help with accurate diagnosis and management.

In cases of suspected pulmonary aspiration due to nasopharyngeal bleeding, after the endobronchial blood clots and mucus have been thoroughly removed, sugammadex can be used to help patients fully recover from muscle relaxation and cough up any remaining blood from the airways. Sugammadex is a recently developed, specific reversal agent for steroidal neuromuscular blocking agents (NMBAs). Sugammadex forms an inclusion complex by encapsulating the steroidal NMBA, thereby eliminating the NMBA from its effect site in the neuromuscular junction. The most commonly used agents for NMB reversal are acetylcholinesterase inhibitors, which prevent the breakdown of acetylcholine in the neuromuscular junction, thereby increasing neuromuscular transmission. However, sugammadex has been reported to have several benefits over acetylcholinesterase inhibitors, including its ability to rapidly and predictably reverse any degree of NMB and prevent recurarization.^[17]^ Compared with sugammadex, acetylcholinesterase inhibitors have a slower onset of action and they exhibit a ceiling effect when used in large doses; therefore, acetylcholinesterase inhibitors can result in inadequate reversal of deep NMB. Recent studies have recommended that acetylcholinesterase inhibitors only be administered after a fourth twitch of TOF stimulation is detectable.^[6,7,18]^ Furthermore, even when a fourth twitch is detectable, the use of acetylcholinesterase inhibitors cannot guarantee successful NMB reversal in patients with a TOF ratio greater than 0.9.^[6]^ Therefore, sugammadex is recommended in cases in which intubation or ventilation is difficult after administration of a muscle relaxant or when a difficult airway is anticipated, necessitating complete postoperative recovery from muscle relaxation. The patient in the present case showed regurgitation of blood remaining in the bronchi and nasopharynx even after extubation. Therefore, if the muscle relaxation had not been completely reversed and the patient was incapable of active coughing, this mucus and blood could have been re-aspirated into the trachea, and the patient could have become dangerously hypoxemic.

In conclusion, patients with oropharyngeal and nasopharyngeal bleeding should be closely monitored for blood aspiration throughout the perioperative period. If blood aspiration occurs, the development of severe pulmonary complications can be prevented by using an FOB to actively remove the aspirated blood. Moreover, sugammadex can be administered to ensure complete reversal of muscle relaxation and enable patients to cough up any blood clots and mucus remaining in the airways.

## Author contributions

**Conceptualization:** Taeha Ryu, Sung Hye Byun.

**Writing – original draft:** Taeha Ryu, Dong Hyuck Kim, Sung Hye Byun.

**Writing – review and editing:** Sung Hye Byun.
